# Severe renal and pancreatic toxicities associated with ipilimumab and nivolumab combination therapy in non-small cell lung cancer: a pharmacovigilance analysis of the FDA adverse event reporting system

**DOI:** 10.3389/fimmu.2026.1854177

**Published:** 2026-06-17

**Authors:** Jiongrui Cao, Li Peng, Jin Yang, Gang Yin, Ping Ke, Jifa Zhang, Min Su, Yun Gao, Yuehui Zhang

**Affiliations:** 1 North Sichuan Medical College, Nanchong, Sichuan, China; 2 Dali University, Dali, Yunnan, China; 3 Department of Oncology, Panzhihua Central Hospital, Panzhihua, Sichuan, China

**Keywords:** glomerulonephritis, immune-related adverse events, ipilimumab, nephrotoxicity, nivolumab, non-small cell lung cancer, pancreatic toxicity, pharmacovigilance

## Abstract

**Background:**

Ipilimumab plus nivolumab (Ipi+Nivo) has become a standard therapy for advanced non-small cell lung cancer (NSCLC), yet severe organ-specific toxicities remain inadequately characterized. This study aimed to characterize severe renal and pancreatic toxicities associated with Ipi+Nivo combination therapy versus nivolumab monotherapy in NSCLC patients.

**Methods:**

We analyzed the FDA Adverse Event Reporting System (FAERS) database from 2004 Q1 to 2025 Q2. Disproportionality analysis using reporting odds ratio (ROR) and information component (IC) identified safety signals. Time-to-onset and clinical severity were compared between treatment groups.

**Results:**

Among 2,292 reports, combination therapy demonstrated significant disproportionality signals for glomerular-predominant renal injury, including glomerulonephritis minimal lesion (ROR = 78.62, IC025 = 2.096), nephrotic syndrome (ROR = 37.25, IC025 = 1.914), and glomerulosclerosis (ROR = 61.79, IC025 = 2.015). Pancreatic toxicity showed strong signals (ROR = 61.79, IC025 = 2.015). Additional renal signals included nephritis (ROR = 24.99, IC025 = 1.832) and renal failure (ROR = 2.99, IC025 = 0.499). Among 447 combination therapy reports, 39 renal events and 23 pancreatic events were identified, compared to 114 and 48 events respectively in 1,814 monotherapy reports. Median time-to-onset was 73 days for renal events and 84 days for pancreatic events in the combination group. Co-reported renal and pancreatic events were infrequent at the report level (2 of 60 combination therapy reports with renal and/or pancreatic events), and serious clinical outcomes including death and hospitalization were prevalent in both groups without statistically significant between-group differences.

**Conclusions:**

Ipi+Nivo combination therapy is associated with prominent glomerular-predominant renal injury and pancreatic toxicity signals, potentially reflecting distinct immunological mechanisms whereby CTLA-4-dependent disruption of B-cell tolerance may predispose to glomerular injury while PD-1-mediated release of T-cell cytotoxicity may preferentially target pancreatic islets. These findings suggest the need for enhanced clinical vigilance and tailored monitoring strategies beyond conventional acute interstitial nephritis surveillance.

## Introduction

Lung cancer remains the most frequently diagnosed malignancy and the leading cause of cancer-related mortality worldwide, with approximately 2.4 million new cases and nearly 1.8 million deaths reported annually (1). Non-small cell lung cancer (NSCLC) accounts for more than 85% of all lung cancer diagnoses and continues to impose a substantial clinical and public health burden due to its high prevalence, frequent presentation at advanced stages, and limited long-term survival (2). In China, lung cancer remained the most commonly diagnosed malignancy in 2022, with approximately 1.06 million new cases and more than 730,000 deaths reported nationwide (3). Despite declining incidence trends in some high-income countries, population ageing, environmental exposures, and expanded screening have contributed to a persistently high global burden of NSCLC (1, 4).

Immune checkpoint inhibitors (ICIs) have become an integral component of systemic therapy for advanced NSCLC. In particular, dual immune checkpoint blockade with ipilimumab, a cytotoxic T-lymphocyte–associated antigen 4 inhibitor, and nivolumab, a programmed death 1 inhibitor, has demonstrated durable survival benefits compared with chemotherapy alone, with long-term follow-up confirming sustained overall survival advantages (5, 6). However, these therapeutic gains are accompanied by an increased burden of immune-related adverse events, as combination immune checkpoint blockade has been consistently associated with a broader spectrum and greater severity of toxicity than PD-1 inhibitor monotherapy (5, 6).

Through concurrent blockade of cytotoxic T-lymphocyte-associated antigen 4 and programmed death 1 pathways, ipilimumab and nivolumab enhance antitumor T-cell immunity while altering immune tolerance, thereby predisposing patients to immune-related adverse events (7). While dermatologic, gastrointestinal, and endocrine toxicities are most frequently reported, renal and pancreatic toxicities are less common and may be underrecognized in routine clinical practice (8). Immune-related acute kidney injury most commonly presents as acute interstitial nephritis and may result in incomplete renal recovery if not promptly recognized (9), whereas immune-mediated pancreatic injury, particularly immune checkpoint inhibitor-induced type 1 diabetes mellitus, is characterized by rapid and irreversible β-cell destruction and may present with diabetic ketoacidosis, leading to long-term metabolic consequences (10).

Evidence on renal and pancreatic toxicities associated with immune checkpoint inhibitors is largely derived from case reports, small retrospective cohorts, and narrative reviews. Available data indicate that renal toxicity typically manifests as acute interstitial nephritis (11), whereas pancreatic toxicity most often presents as immune-mediated pancreatitis or type 1 diabetes mellitus (12). In contrast, glomerular-predominant renal injury has been reported only sporadically, and pancreatic toxicity patterns associated with ipilimumab plus nivolumab remain insufficiently characterized.

To address this gap, we analyzed data from the U.S. Food and Drug Administration Adverse Event Reporting System (FAERS), a large-scale pharmacovigilance database that captures spontaneous adverse event reports using standardized Medical Dictionary for Regulatory Activities terminology (7, 13). Using disproportionality analysis, we examined reported severe renal and pancreatic toxicities associated with ipilimumab plus nivolumab in patients with NSCLC, compared with nivolumab monotherapy, and characterized differences in reporting signals, time to onset, and clinical severity.

## Methods

### Data source

The FAERS database is a publicly accessible spontaneous reporting system maintained by the U.S. Food and Drug Administration to support post-marketing drug safety surveillance. Data were extracted from the FAERS database spanning the first quarter of 2004 through the second quarter of 2025 (14). Duplicate reports were removed following FDA recommendations: when case identification number (CASEID) was identical, we selected the report with the latest FDA receipt date (FDA_DT); when both CASEID and FDA_DT were identical, we retained the record with the higher primary identification number (PRIMARYID). This study utilized publicly available, de-identified data and was conducted in accordance with the Declaration of Helsinki. As this analysis involved retrospective examination of de-identified data from a publicly available database without direct patient contact, this study was exempt from institutional review board approval and informed consent requirements.

### Study population and cohort definition

Three treatment cohorts were defined based on drug role codes (Primary Suspect or Secondary Suspect): the combination therapy group included reports listing both nivolumab and ipilimumab concurrently; the nivolumab monotherapy group comprised reports with nivolumab alone without ipilimumab; and the ipilimumab monotherapy group contained reports with ipilimumab alone without nivolumab, serving as an auxiliary comparator. Reports were restricted to non-small cell lung cancer (NSCLC) indications, identified through MedDRA preferred terms (PTs) including “non-small cell lung cancer”, “NSCLC”, “lung adenocarcinoma”, and “lung squamous cell carcinoma”.

### Adverse event definition

Renal and pancreatic adverse events were identified using MedDRA version 26.0 PTs. Renal toxicities were classified into four pathophysiological categories based on the primary site of injury: glomerular injury (including glomerulonephritis minimal lesion, nephrotic syndrome, and glomerulosclerosis), tubular injury (including renal tubular injury and tubulointerstitial nephritis), vascular injury (including renal arteriosclerosis), and general renal dysfunction (including acute kidney injury, renal failure, nephritis, blood creatinine increased, and renal impairment). Pancreatic toxicities were categorized into three functional domains: exocrine pancreatic injury (including pancreatic toxicity and pancreatitis), pancreatic enzyme elevation (including lipase increased and amylase increased), and endocrine pancreatic dysfunction (including diabetic ketoacidosis, type 1 diabetes mellitus, hyperglycaemia, and diabetes mellitus). This classification system was designed to capture both acute inflammatory events and chronic metabolic sequelae associated with immune-mediated pancreatic injury. It should be noted that the adverse event terms extracted in this analysis strictly correspond to MedDRA-defined standard Preferred Terms. Because real-world pharmacovigilance reports are submitted by diverse sources, the granularity of reported terms varies considerably: specialist physicians may submit highly specific histopathologic diagnoses (e.g., “glomerulonephritis minimal lesion”), whereas general practitioners or consumer reporters may use broader symptomatic descriptors (e.g., “nephritis”). This inherent variability in reporting specificity is a recognized characteristic of the FAERS coding system and should be considered when interpreting the clinical precision of individual PT-level signals.

### Statistical analysis

Disproportionality analysis was performed to detect potential safety signals by comparing the reporting frequency of specific adverse events between the combination therapy group and the nivolumab monotherapy group (15). The ROR was calculated as ROR = (a/b)/(c/d), where a represents the number of target adverse events in the combination group, b represents the number of all other adverse events in the combination group, c represents the number of target adverse events in the nivolumab monotherapy group, and d represents the number of all other adverse events in the nivolumab monotherapy group. The 95% confidence interval (CI) of ROR was calculated using the standard error of the natural logarithm of ROR. The information component (IC) and its lower 95% confidence bound (IC025) were computed as Bayesian measures of disproportionality. A positive signal was defined when all three criteria were met: the lower bound of the 95% CI of ROR exceeded 1.0 (ROR025 > 1), IC025 was greater than 0, and the false discovery rate (FDR)-adjusted p-value was less than 0.05, with a minimum of three reported cases (16). To address zero-cell counts in the contingency tables for rare events, the Haldane-Anscombe correction was applied by adding 0.5 to all cells prior to ROR calculation. Multiple testing correction was performed using the Benjamini-Hochberg procedure to control the false discovery rate across all tested PTs. Time-to-onset (TTO) was defined as the interval between treatment initiation (or FDA receipt date when treatment start date was unavailable) and adverse event occurrence. Median TTO and interquartile ranges were calculated for each event category. To characterize the clinical severity of reported events, the proportions of different FDA-defined serious outcomes (death, life-threatening, hospitalization, disability, and other serious outcomes) were calculated and described for renal and pancreatic toxicities within the combination therapy and nivolumab monotherapy groups. To further characterize the clinical profile of affected patients, supplementary analyses were performed. Overlap analysis identified reports with co-reported renal and pancreatic adverse events within each treatment cohort using unique report identifiers after deduplication. Histologic subtypes of NSCLC were extracted from the indications (INDI) table by searching for specific pathologic terms (e.g., lung adenocarcinoma, lung squamous cell carcinoma) among patients with targeted adverse events. Comorbidity-related clinical context was approximated using non-cancer indications and indications associated with co-administered medications, after excluding oncologic indications and the target adverse event terms themselves. Differences in serious clinical outcomes between treatment groups were assessed using Fisher exact test. All statistical analyses were performed using Python 3.10 with pandas, scipy.stats, matplotlib, and seaborn packages.

## Results

### Baseline characteristics of FAERS reports

A total of 2,292 adverse event reports were included, comprising 447 reports in the ipilimumab plus nivolumab combination group, 1,814 reports in the nivolumab monotherapy group, and 31 reports in the ipilimumab monotherapy group. The mean age was 65.4 ± 9.7 years in the combination group, 66.0 ± 9.8 years in the nivolumab monotherapy group, and 67.8 ± 6.3 years in the ipilimumab monotherapy group. The median age was 67.0 (IQR: 60.0–72.0) years in the combination group, 67.0 (60.0–73.0) years in the nivolumab monotherapy group, and 65.5 (65.0–68.0) years in the ipilimumab monotherapy group. Missing age information was reported in 26 (5.8%), 234 (12.9%), and 19 (61.3%) cases, respectively. Male patients accounted for 264 (59.1%) reports in the combination group, 1,127 (62.1%) in the nivolumab monotherapy group, and 8 (25.8%) in the ipilimumab monotherapy group. Female patients accounted for 168 (37.6%), 603 (33.2%), and 5 (16.1%) reports, respectively, with unknown or missing sex reported in 15 (3.4%), 84 (4.6%), and 17 (54.8%) cases. Reports from 2021 to 2025 accounted for 194 (43.4%) cases in the combination group, 222 (12.2%) in the nivolumab monotherapy group, and 3 (9.7%) in the ipilimumab monotherapy group. The most frequently reported countries in the combination group were the United States (118, 26.4%), Japan (111, 24.8%), and Italy (45, 10.1%), whereas in the nivolumab monotherapy group the most frequent reporting country was France (541, 29.8%). In the ipilimumab monotherapy group, 24 reports (77.4%) originated from the United States. Healthcare professionals submitted 376 (84.1%) reports in the combination group, 1,230 (67.8%) in the nivolumab monotherapy group, and 17 (54.8%) in the ipilimumab monotherapy group. Regarding serious outcomes, death was reported in 76 (17.0%) cases in the combination group, 374 (20.6%) cases in the nivolumab monotherapy group, and 14 (45.2%) cases in the ipilimumab monotherapy group. Life-threatening outcomes were reported in 57 (12.8%), 148 (8.2%), and 2 (6.5%) cases, respectively. Hospitalization was reported in 216 (48.3%) combination therapy cases, 849 (46.8%) nivolumab monotherapy cases, and 11 (35.5%) ipilimumab monotherapy cases. Disability was reported in 4 (0.9%) and 49 (2.7%) cases in the combination and nivolumab monotherapy groups, respectively. Other serious outcomes were reported in 384 (85.9%) combination therapy cases, 1,460 (80.5%) nivolumab monotherapy cases, and 7 (22.6%) ipilimumab monotherapy cases (**Table 1**).

**Table 1:** Baseline characteristics of FAERS adverse event reports across treatment groups.

Variable	Combination (n=447)	Nivolumab Mono (n=1, 814)
Age, years	65.4 ± 9.7	66.0 ± 9.8
Median (IQR)	67.0 (60.0-72.0)	67.0 (60.0-73.0)
Missing, n (%)	26 (5.8%)	234 (12.9%)
Sex, n (%)		
Male	264 (59.1%)	1127 (62.1%)
Female	168 (37.6%)	603 (33.2%)
Unknown/Missing	15 (3.4%)	84 (4.6%)
Report Year, n (%)		
2010-2015	0 (0.0%)	78 (4.3%)
2016-2020	75 (16.8%)	779 (42.9%)
2021-2025	194 (43.4%)	222 (12.2%)
Reporter Country, n (%)		
FR	18 (4.0%)	541 (29.8%)
US	118 (26.4%)	398 (21.9%)
JP	111 (24.8%)	195 (10.7%)
IT	45 (10.1%)	143 (7.9%)
DE	16 (3.6%)	84 (4.6%)
Other/Unknown	139 (31.1%)	453 (25.0%)
Reporter Type, n (%)		
Healthcare Professional	376 (84.1%)	1230 (67.8%)
Consumer	34 (7.6%)	246 (13.6%)
Other/Unknown	37 (8.3%)	338 (18.6%)
Serious Outcome, n (%)		
Death	76 (17.0%)	374 (20.6%)
Life-threatening	57 (12.8%)	148 (8.2%)
Hospitalization	216 (48.3%)	849 (46.8%)
Disability	4 (0.9%)	49 (2.7%)
Other Serious	384 (85.9%)	1460 (80.5%)

Data presented as n (%) for categorical variables and mean ± SD [median (IQR)] for continuous variables. No statistical testing was performed as p-values are inappropriate for spontaneous reporting system (SRS) data due to inherent reporting biases and large sample sizes that render statistical significance misleading.

### Signal detection of renal toxicities

Signal detection analysis comparing nivolumab plus ipilimumab combination therapy with nivolumab monotherapy identified multiple renal toxicity signals meeting predefined criteria (ROR lower bound > 1, IC025 > 0, and FDR-adjusted P < 0.05). Within the glomerular injury category, significant signals were observed for glomerulonephritis minimal lesion (9 vs. 0 cases; ROR = 78.62, 95% CI: 4.57–1353.46; IC025 = 2.096; P < 0.001), nephrotic syndrome (9 vs. 1 case; ROR = 37.25, 95% CI: 4.71–294.83; IC025 = 1.914; P < 0.001), and glomerulosclerosis (7 vs. 0 cases; ROR = 61.79, 95% CI: 3.52–1083.94; IC025 = 2.015; P < 0.001). For tubular injury, renal tubular injury showed a significant signal (7 vs. 0 cases; ROR = 61.79, 95% CI: 3.52–1083.94; IC025 = 2.015; P < 0.001). Within the vascular injury category, renal arteriosclerosis demonstrated a positive signal (6 vs. 0 cases; ROR = 53.43, 95% CI: 3.00–950.24; IC025 = 1.959; P < 0.001). Regarding general renal dysfunction, significant associations were observed for renal failure (13 vs. 18 cases; ROR = 2.99, 95% CI: 1.45–6.15; IC025 = 0.499; P = 0.006) and nephritis (12 vs. 2 cases; ROR = 24.99, 95% CI: 5.57–112.08; IC025 = 1.832; P < 0.001) (**Table 2**).

**Table 2:** Signal detection results of renal toxicities associated with nivolumab plus ipilimumab versus nivolumab monotherapy in NSCLC patients.

Category/PT	Combo cases	Mono cases	ROR (95% CI)	IC025	FDR p-value
Glomerular Injury					
Glomerulonephritis minimal lesion	9	0	78.62 (4.57-1353.46)	2.096	<0.001
Nephrotic syndrome	9	1	37.25 (4.71-294.83)	1.914	<0.001
Glomerulosclerosis	7	0	61.79 (3.52-1083.94)	2.015	<0.001
Tubular Injury					
Renal tubular injury	7	0	61.79 (3.52-1083.94)	2.015	<0.001
Tubulointerstitial nephritis	4	19	0.85 (0.29-2.52)	-1.464	0.758
Vascular Injury					
Renal arteriosclerosis	6	0	53.43 (3.00-950.24)	1.959	<0.001
General Renal Dysfunction					
Acute kidney injury	15	49	1.25 (0.69-2.25)	-0.382	0.326
Renal failure	13	18	2.99 (1.45-6.15)	0.499	0.006
Nephritis	12	2	24.99 (5.57-112.08)	1.832	<0.001
Blood creatinine increased	7	11	2.61 (1.01-6.77)	0.149	0.072
Renal impairment	6	13	1.88 (0.71-4.99)	-0.272	0.208
Renal disorder	1	6	0.68 (0.08-5.63)	-3.084	0.787

PT, Preferred Term; ROR, Reporting Odds Ratio; CI, Confidence Interval; IC025, Information Component lower bound (2.5th percentile); FDR, False Discovery Rate. A signal is considered positive if ROR lower bound >1, IC025 >0, and FDR p-value <0.05.

### Signal detection of pancreatic and endocrine-pancreatic toxicities

Signal detection analysis identified a significant pancreatic toxicity signal for nivolumab plus ipilimumab combination therapy compared with nivolumab monotherapy. Within the exocrine pancreatic injury category, pancreatic toxicity showed a strong disproportionality signal (7 vs. 0 cases; ROR = 61.79, 95% CI: 3.52–1083.94; IC025 = 2.015; P < 0.001), meeting all predefined signal detection criteria. No other pancreatic enzyme elevation or endocrine-pancreatic dysfunction terms satisfied the signal thresholds (**Table 3**).

**Table 3:** Signal detection results of pancreatic and endocrine-pancreatic toxicities associated with nivolumab plus ipilimumab versus nivolumab monotherapy in NSCLC patients.

Category/PT	Combo cases	Mono cases	ROR (95% CI)	IC025	FDR p-value
Exocrine Pancreatic Injury					
Pancreatic toxicity	7	0	61.79 (3.52-1083.94)	2.015	<0.001
Pancreatitis	2	12	0.67 (0.15-3.03)	-2.316	0.829
Pancreatic Enzyme Elevation					
Lipase increased	3	2	6.12 (1.02-36.75)	0.576	0.149
Amylase increased	1	1	4.07 (0.25-65.12)	-0.657	0.475
Endocrine Pancreatic Dysfunction (Diabetes)					
Diabetic ketoacidosis	6	10	2.45 (0.89-6.79)	0.019	0.153
Type 1 diabetes mellitus	5	6	3.41 (1.04-11.22)	0.275	0.149
Hyperglycaemia	3	3	4.08 (0.82-20.28)	0.190	0.153
Diabetes mellitus	1	7	0.58 (0.07-4.72)	-3.304	0.829

PT, Preferred Term; ROR, Reporting Odds Ratio; CI, Confidence Interval; IC025, Information Component lower bound (2.5th percentile); FDR, False Discovery Rate. A signal is considered positive if ROR lower bound >1, IC025 >0, and FDR p-value <0.05. Endocrine pancreatic dysfunction (diabetes) is integrated into this table as ICI-related pancreatic injury often manifests as pancreatitis-induced diabetes or autoimmune diabetes.

### Report-level overlap and clinical-context analyses

Overlap analysis using unique report identifiers revealed that renal and pancreatic adverse events were infrequently co-reported in both treatment cohorts. In the combination therapy group, 39 reports involved renal events and 23 involved pancreatic events; among 60 unique reports with renal and/or pancreatic events, 2 reports included both organ toxicities. In the nivolumab monotherapy group, 7 co-reported both renal and pancreatic events among 114 reports with renal events and 48 reports with pancreatic events (**Supplementary Table 1**).

Among reports with renal or pancreatic adverse events and available histology-related indication terms in the INDI table, squamous cell carcinoma of the lung was the most frequently documented term in both the combination therapy group (54 cases) and the nivolumab monotherapy group (73 cases). Large cell lung cancer was identified in one nivolumab monotherapy report. Most affected reports lacked specific histologic subtype documentation beyond the general NSCLC indication, reflecting the limited availability of detailed pathologic information in spontaneous reporting systems (**Supplementary Table 2**).

Proxy analysis of non-cancer indication terms provided descriptive clinical context across treatment groups. In the combination therapy group, the most frequently documented non-cancer indication proxies were urinary tract infection, prophylaxis, antibiotic therapy, chronic cardiac failure, hypertension, and depression. In the nivolumab monotherapy group, hypertension, atrial fibrillation, and gastroesophageal reflux disease were the most frequently documented non-cancer indication proxies (**Supplementary Table 3**).

### Severity distribution of renal and pancreatic adverse events

**Figure 1:**
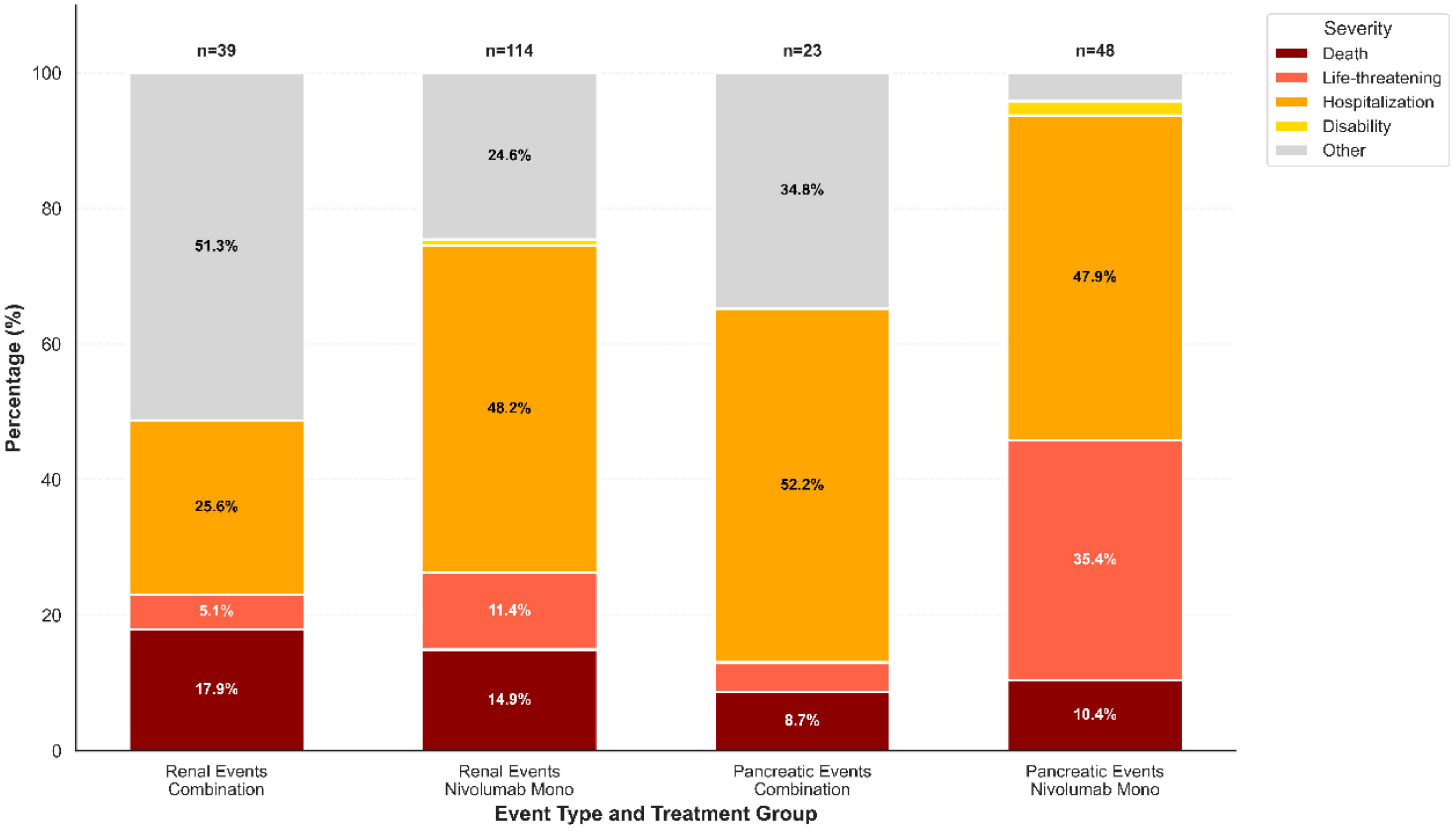
Severity distribution of reported renal and pancreatic adverse events in FAERS reports with NSCLC indications for nivolumab plus ipilimumab versus nivolumab monotherapy.

For renal events, the combination therapy group (n = 39) showed proportions of death 17.9%, life-threatening events 5.1%, hospitalization 25.6%, and other serious outcomes 51.3%. In the nivolumab monotherapy group (n = 114), death accounted for 14.9%, life-threatening events for 11.4%, hospitalization for 48.7%, and other serious outcomes for 24.6%. For pancreatic events, the combination therapy group (n = 23) exhibited death in 8.7%, life-threatening events in 4.3%, hospitalization in 52.2%, and other serious outcomes in 34.8%. In the nivolumab monotherapy group (n = 48), death accounted for 10.4%, life-threatening events for 35.4%, hospitalization for 47.9%, and other serious outcomes for 6.3% (**Figure 1**). When renal and pancreatic events were pooled at the report level, serious outcome codes were common in both groups: death was recorded in 7 of 60 patients (11.7%) in the combination therapy group and 21 of 156 patients (13.5%) in the nivolumab monotherapy group (P = 0.824); hospitalization was reported in 32 (53.3%) and 106 (67.9%) patients, respectively (P = 0.058). No statistically significant differences in the proportions of death, life-threatening events, hospitalization, or disability were observed between the two groups (**Supplementary Table 4**). Although the between-group differences in outcome proportions did not reach statistical significance, the high overall prevalence of serious outcomes in both cohorts underscores the substantial clinical burden of these toxicities regardless of treatment regimen.

### Time-to-onset and clinical outcome analysis of renal and pancreatic toxicities

**Figure 2:**
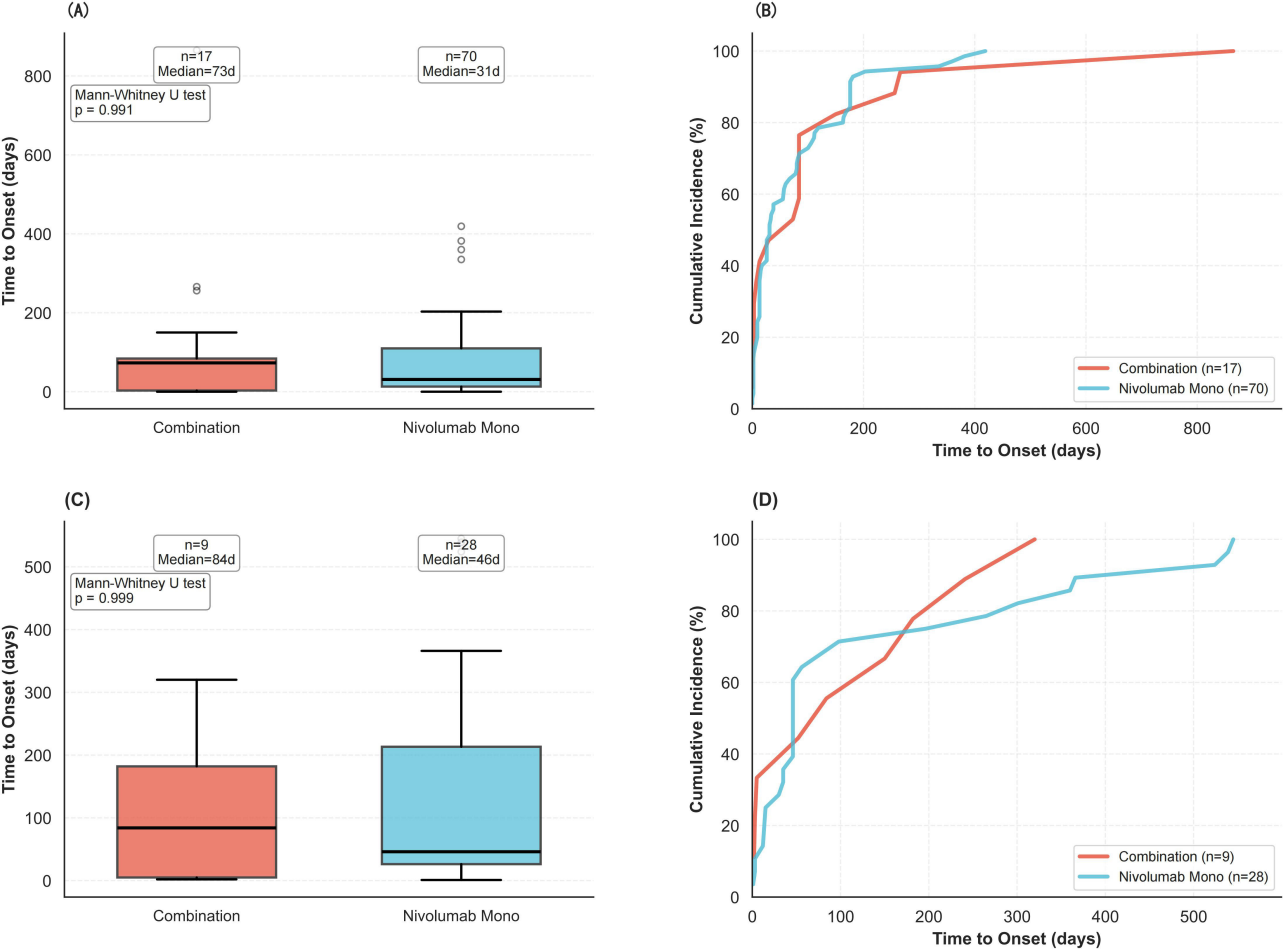
Time-to-onset distribution and cumulative number of reports of renal and pancreatic adverse events in NSCLC patients treated with nivolumab plus ipilimumab versus nivolumab monotherapy. **(A)** Renal Events; **(B)** Renal Events - Cumulative Incidence; **(C)** Pancreatic Events; **(D)** Pancreatic Events - Cumulative Incidence.

For renal adverse events, time-to-onset distributions and cumulative incidence patterns are presented in **Figures 2A**, **B**, with sample sizes of n = 17 in the combination group and n = 70 in the monotherapy group. The median reported time to onset was 73 days (IQR: 3–84) in the combination therapy group and 31 days (IQR: 13–110) in the nivolumab monotherapy group. Fatal renal events were reported in 6 cases (30.0%) in the combination group and 14 cases (15.9%) in the monotherapy group. For pancreatic adverse events (**Figures 2C**, **D**), the median reported time to onset was 84 days (IQR: 5–182; n = 9) in the combination therapy group and 46 days (IQR: 26–213; n = 28) in the nivolumab monotherapy group (**Table 4**). Fatal pancreatic events occurred in 2 cases (12.5%) in the combination group and 2 cases (5.1%) in the monotherapy group. The cumulative incidence curves showed early event accumulation patterns in both treatment groups.

**Table 4:** Time-to-onset characteristics and fatal outcomes of renal and pancreatic adverse events in NSCLC patients treated with nivolumab plus ipilimumab versus nivolumab monotherapy.

Event type	Metric	Combination	Nivolumab mono
Renal Events	Median Time to Onset (Days) [IQR]	73 [3-84] (n=17)	31 [13-110] (n=70)
	Fatality Rate, n (%)	6 (30.0%)	14 (15.9%)
Pancreatic Events	Median Time to Onset (Days) [IQR]	84 [5-182] (n=9)	46 [26-213] (n=28)
	Fatality Rate, n (%)	2 (12.5%)	2 (5.1%)

IQR, Interquartile Range. Time to onset calculated as days from treatment initiation (start_dt) or initial FDA receipt date (init_fda_dt) to event date. Fatality rate defined as proportion of cases with death outcome (outc_cod=‘DE’). For pancreatic events, fatal outcomes occurred in 2 cases (12.5%) in the combination group and 2 cases (5.1%) in the monotherapy group.

## Discussion

The present pharmacovigilance analysis suggests that dual checkpoint blockade with ipilimumab plus nivolumab in non-small cell lung cancer reports a distinctive safety profile that concentrates on severe, glomerular-predominant kidney injury and clinically consequential pancreatic toxicity, rather than the more familiar interstitial pattern emphasized in routine monitoring. The clinical value of this signal lies in raising awareness and generating hypotheses for prospective validation: these findings suggest that glomerular injury and pancreatic toxicity may warrant increased clinical suspicion during combination therapy, potentially complementing existing surveillance frameworks that primarily focus on creatinine-based acute interstitial nephritis detection.

Related research suggests that kidney immune-related adverse events have often been framed around acute interstitial nephritis, partly because early clinicopathologic series and guideline discussions focused on creatinine rises and biopsy patterns dominated by interstitial inflammation. More recent syntheses, however, have collected an expanding set of biopsy-proven glomerular lesions linked to checkpoint inhibitors, including podocytopathies and focal segmental glomerulosclerosis, sometimes overlapping with interstitial disease rather than replacing it (17). Reviews of checkpoint inhibitor nephrotoxicity have also highlighted that combination regimens tend to broaden the renal phenotype and may increase the likelihood that atypical presentations prompt nephrology evaluation and biopsy, which in turn reshapes what gets recognized and reported (18, 19). The 2024 position statement from the American Society of Onco-nephrology further affirmed that glomerular disease constitutes a recognized component of the checkpoint inhibitor-associated kidney injury spectrum beyond the predominant acute interstitial nephritis pattern (20). Against that backdrop, the concentration of disproportionality signals in glomerular diagnostic terms supports a shift in what clinicians should look for when kidney injury develops during combination therapy, even when creatinine changes appear modest at first (17–19).

A glomerular-centered signal also fits with mechanistic proposals that place podocytes and the glomerular filtration barrier among plausible immune targets once peripheral tolerance loosens. Case-based literature has described nephrotic presentations during PD-1 blockade with biopsy patterns such as focal segmental glomerulosclerosis and minimal change disease, and several reports required treatment escalation beyond corticosteroids, suggesting that the inflammatory program can persist after drug withdrawal in at least some patients (21, 22). Broader compilations of checkpoint inhibitor–associated glomerular disease have likewise described podocytopathy as a recurring lesion class, frequently accompanied by heavy proteinuria and hypoalbuminemia, features that routine creatinine surveillance can miss (17). In practical terms, the “renal event” most worth fearing in this setting may not be an abrupt creatinine spike but a proteinuric syndrome that evolves quietly until edema, thrombotic risk, or infection risk forces urgent care (17, 21, 22).

The immunological basis for this glomerular-predominant pattern merits consideration. CTLA-4 and PD-1 operate at different stages of the immune response: CTLA-4 primarily modulates T-cell priming and is constitutively expressed on regulatory T cells, whereas PD-1 restrains effector T-cell function in peripheral tissues (23). Dual blockade therefore not only amplifies T-cell activation but may qualitatively alter the immune response by simultaneously expanding effector repertoires and impairing regulatory T-cell-mediated suppression (23, 24). Such Treg dysfunction may weaken local self-tolerance at the glomerular filtration barrier, thereby providing a plausible immunological pathway linking dual checkpoint blockade to podocyte injury and proteinuric glomerular disease. Critically, recent evidence has demonstrated that CTLA-4 blockade, but not PD-1 blockade alone, disrupts peripheral B-cell tolerance and promotes the emergence of autoreactive mature naive B cells targeting self-antigens expressed in tissues affected by immune-related adverse events (25). This CTLA-4-dependent breach in humoral tolerance provides a mechanistic link to antibody-mediated and immune complex-mediated glomerular disease. Podocytes, which are now recognized to express complement components and share properties of innate immunity, may serve as both sources and targets of complement-mediated injury under such conditions of dysregulated autoimmunity (26). A recent case of nephrotic syndrome following ipilimumab plus nivolumab, with biopsy-confirmed minimal change disease and near-complete podocyte foot process effacement concurrent with acute interstitial nephritis, illustrates that combination blockade can produce a mixed glomerular-interstitial phenotype undetectable through creatinine monitoring alone (27).

Pancreatic toxicity in checkpoint inhibitor practice has remained harder to pin down because the phenotype ranges from incidental enzyme elevations to symptomatic pancreatitis and endocrine failure. Cohort work in immunotherapy-treated populations has shown that pancreatic injury may progress to chronic sequelae, including diabetes and pancreatic structural changes, while clinical symptoms remain variable and imaging can lag behind biochemistry (28). A more recent synthesis that paired systematic review methods with real-world pharmacovigilance analysis reinforced that pancreatitis and pancreatic injury appear uncommon but clinically meaningful, and that diagnosis often hinges on excluding alternative causes rather than on any single definitive marker (29). Taken together, those observations make it plausible that spontaneous reports coded under composite pancreatic terms capture a heterogeneous set of injuries rather than one uniform disease entity, which matters when translating a reporting signal into bedside monitoring priorities (28, 29).

The immunological distinction between exocrine and endocrine pancreatic injury is relevant to interpreting these signals. Exocrine pancreatic injury may involve cytotoxic T-cell-mediated inflammation in pancreatic tissue, with biopsy-based and translational evidence suggesting infiltration of cytolytic lymphocytes in some immune checkpoint inhibitor-associated pancreatic injury phenotypes (30, 31). Endocrine injury involves more targeted β-cell destruction that parallels classical type 1 diabetes mellitus but has distinctive clinical and immunological features. Available evidence suggests that disruption of the PD-1/PD-L1 axis may permit autoreactive CD8+ T-cell-mediated β-cell injury, with genetic susceptibility, including HLA background, potentially influencing individual risk (32–34). In animal models, anti-PD-L1 but not anti-CTLA-4 monotherapy rapidly induced diabetes through cytolytic interferon-gamma-positive CD8+ T-cell islet infiltration (31). This PD-1-dominant mechanism in endocrine injury contrasts with the CTLA-4-dependent B-cell tolerance disruption implicated in glomerular disease (25), suggesting that different components of dual blockade may preferentially contribute to distinct organ-specific toxicities.

Endocrine pancreatic dysfunction deserves separate emphasis because the metabolic trajectory does not necessarily track the severity of abdominal symptoms. Reviews of checkpoint inhibitor–related diabetes describe a pattern of abrupt insulin deficiency that can present with ketoacidosis and persists even after immunotherapy cessation, implying rapid β-cell destruction rather than a transient inflammatory stress response (33, 34). Unlike classical type 1 diabetes, checkpoint inhibitor-associated diabetes typically manifests after only a few treatment cycles, presents with disproportionately modest HbA1c elevation, and is generally glucocorticoid-refractory (32). The clinical narrative emerging from this literature places combination checkpoint blockade among the regimens that warrant heightened suspicion, particularly when patients develop polyuria, weight loss, confusion, or intercurrent illness that destabilizes glucose control. In that context, the concept of concurrent glomerular and pancreatic vulnerability should be interpreted as a working hypothesis rather than a confirmed syndromic entity. The overlap analysis in this study identified co-reported renal and pancreatic events in only 2 of 60 combination therapy reports with renal and/or pancreatic events, indicating that report-level co-occurrence was infrequent. Nevertheless, both organ systems depend on local immune tolerance mechanisms disrupted through partially distinct pathways during dual blockade: CTLA-4 blockade predominantly contributes to humoral autoimmunity targeting the glomerular filtration barrier (25, 26), whereas PD-1 blockade preferentially releases CD8+ T-cell cytotoxicity against PD-L1-expressing pancreatic islets (31, 32). Whether shared effector populations or overlapping cytokine environments contribute to concurrent injury in a subset of patients remains to be determined through prospective mechanistic studies.

Guidance documents for immune-related adverse events already recommend routine laboratory surveillance and prompt workup of suspected nephritis and endocrine toxicity, but the glomerular and pancreatic themes highlighted here argue for more targeted, testable additions to that baseline. Current oncology guidelines generally support regular renal function checks during immunotherapy and early specialist input when kidney injury is suspected (35, 36). For combination therapy in particular, urinalysis should not serve as an optional extra ordered only after creatinine rises; it can function as an early warning system when it includes dipstick protein screening followed by quantitative protein assessment and urine sediment review if abnormalities appear (35, 36). On the pancreatic side, relying only on symptom-triggered testing risks missing abrupt insulin deficiency; fasting or random glucose checks integrated into routine treatment visits, with immediate ketone assessment when hyperglycemia accompanies systemic symptoms, provides a concrete safety net that aligns with how checkpoint inhibitor–related diabetes tends to present (33, 34). These signal patterns may inform future prospective monitoring studies. If validated in controlled cohorts with systematic adjudication, enhanced surveillance strategies could potentially include baseline and interval urinalysis for proteinuria screening and structured glucose assessment, particularly when patients present with compatible clinical features such as edema, unexplained weight loss, or metabolic disturbances. However, specific monitoring intervals and thresholds require validation through prospective studies before implementation in routine practice.

If these signals are confirmed in prospective studies, management implications may differ depending on whether injury appears interstitial, glomerular, or predominantly endocrine. Nephrotoxicity reviews emphasize that biopsy often clarifies the dominant lesion and can prevent overly generic “steroid for all” approaches that overlook podocytopathy, immune complex disease, or vascular lesions that may require tailored immunosuppression and closer relapse surveillance (17–19). Some published nephrotic cases during checkpoint blockade required adjunctive agents and experienced relapse during tapering, a clinical course that supports planning for prolonged monitoring even after apparent improvement (21). For pancreatic injury, cohort evidence indicates that supportive care measures such as careful fluid management may influence downstream outcomes, while the role of corticosteroids remains less definitive than in other immune-related toxicities, particularly when pancreatitis features dominate (28). This heterogeneity argues for a decision pathway that keys off clinical syndrome proteinuric nephrotic picture versus rising creatinine with bland urine versus hyperglycemia with ketosis risk rather than off a single umbrella label of “immune-related toxicity” (21, 28).

Interpretation of disproportionality signals requires restraint even when effect estimates appear striking. FAERS provides breadth and timeliness, but the database does not support incidence estimation, and reporting frequency can rise or fall with publicity, litigation, and changes in clinical awareness (14). Methodological work on the reporting odds ratio underscores that it offers an efficient screening statistic for hypothesis generation rather than a direct measure of risk in treated populations, which is especially relevant for rare events that cluster in medically complex patients (37). Accordingly, the observed PT-level disproportionality signals should be interpreted as reporting-pattern signals rather than evidence of higher absolute incidence or poorer clinical outcomes with combination therapy. The most defensible inference, therefore, is not that combination therapy “causes” a particular lesion, but that the pattern of reports justifies targeted clinical vigilance and motivates confirmatory designs, including prospective registries, biopsy-linked cohorts, and mechanistic sampling that can adjudicate causality and identify susceptible subgroups (14, 37).

Several limitations inherent to FAERS shape how these results should be read. Spontaneous reporting captures suspicion rather than verified diagnosis, and both underreporting and selective reporting can distort the apparent prominence of specific events. Therefore, PT-level signals such as “glomerulonephritis minimal lesion” should be interpreted as coded reporting signals rather than biopsy-confirmed diagnoses in all cases. Case completeness varies widely, and missing data on comorbidities, concomitant nephrotoxic drugs, prior autoimmune disease, baseline renal function, imaging, biopsy confirmation, and treatment details limits clinical interpretation. Although supplementary analyses using co-administered medication indications as proxy measures for baseline comorbidities provided additional clinical context, this approach cannot substitute for direct medical history documentation. Furthermore, the limited availability of detailed histologic subtype information beyond the general NSCLC indication precludes analysis of whether specific tumor subtypes confer differential toxicity risks. Confounding by indication and by disease severity remains difficult to address, particularly in advanced cancer where infection, dehydration, obstruction, and paraneoplastic syndromes can mimic immune-mediated injury. Time-to-onset estimates are subject to substantial bias when treatment start dates are unavailable, as FDA receipt date was used as a surrogate in these cases, potentially introducing systematic measurement error that overestimates true latency periods. Additionally, outcome coding may not reflect longitudinal recovery. Finally, although the overlap analysis identified concurrent renal and pancreatic events in only a small proportion of affected patients (2 of 60 in the combination group), this finding was based on report-level co-occurrence using PRIMARYID matching and does not constitute longitudinal clinical confirmation of a syndromic entity. The low observed overlap may reflect true biological independence of these toxicities at the individual level, but may also be influenced by underreporting, temporal separation of events across different reporting periods, or incomplete capture of multi-organ involvement within single reports. Accordingly, the cross-organ vulnerability concept should be treated as a direction for validation rather than a demonstrated syndromic linkage.

## Conclusion

The FAERS reporting pattern for ipilimumab plus nivolumab in non-small cell lung cancer reveals disproportionate signals for glomerular renal injury and pancreatic involvement that may be underrecognized with conventional creatinine-centered surveillance. The immunological framework suggests that CTLA-4 and PD-1 blockade may contribute to these organ-specific toxicities through partially distinct mechanisms, with CTLA-4-dependent disruption of B-cell tolerance potentially predisposing to glomerular injury and PD-1-mediated release of CD8+ T-cell cytotoxicity preferentially targeting PD-L1-expressing pancreatic islets. While the spontaneous reporting nature of FAERS introduces inherent biases including notoriety effects from recent drug approvals, differential reporting patterns across geographic regions, and heightened awareness from published case reports, the consistency and magnitude of these signals warrant clinical attention. The practical value lies in hypothesis generation for prospective validation: these findings suggest that when patients on combination therapy present with edema, unexplained fatigue, or metabolic disturbances, clinicians should maintain heightened suspicion for atypical renal and pancreatic injury patterns, prompting consideration of urine protein assessment and glucose monitoring beyond routine surveillance. Future prospective cohort studies with systematic adjudication and biopsy confirmation are needed to establish true incidence, validate these signals, and develop evidence-based monitoring protocols.

## Data Availability

The original contributions presented in the study are included in the article/**Supplementary Material**. Further inquiries can be directed to the corresponding author.
