# Supplementary material for: Severe renal and pancreatic toxicities associated with ipilimumab and nivolumab combination therapy in non-small cell lung cancer: a pharmacovigilance analysis of the FDA adverse event reporting system
Source: Front Immunol. 2026 Jun 17;17:1854177. doi: 10.3389/fimmu.2026.1854177 (PMC13318663; doi:10.3389/fimmu.2026.1854177)
Supplement: Supplementary file 1 [file Table1.docx]

**Supplementary Table 1. Overlap analysis of co-reported renal and pancreatic adverse events by treatment cohort**

| **Cohort** | **Reports with Renal AEs (n)** | **Reports with Pancreatic AEs (n)** | **Reports with Concurrent Renal and Pancreatic AEs (n)** |
| --- | --- | --- | --- |
| Combination | 39 | 23 | 2 |
| Nivolumab Monotherapy | 114 | 48 | 7 |

**Note:** Overlap was assessed using deduplicated FAERS report identifiers. Counts represent reports with renal adverse events, pancreatic adverse events, or both. Renal and pancreatic categories are not mutually exclusive.

**Supplementary Table 2. Histology-related indication terms among reports with renal or pancreatic adverse events**

| **Cohort** | **Histologic Subtype** | **Reports (n)** |
| --- | --- | --- |
| Combination | Squamous cell carcinoma of lung | 54 |
| Nivolumab Monotherapy | Squamous cell carcinoma of lung | 73 |
| Nivolumab Monotherapy | Large cell lung cancer | 1 |

**Note:** Histology-related terms were extracted from the FAERS indication table. Because detailed tumor histology is not consistently recorded in FAERS, these counts represent available indication-level information rather than a complete histologic subtype distribution.

**Supplementary Table 3. Non-cancer indication proxies among reports with renal or pancreatic adverse events**

| **Cohort** | **Non-cancer Indication Proxy** | **Cases (n)** |
| --- | --- | --- |
| **Combination** |  |  |
|  | Urinary tract infection | 12 |
|  | Prophylaxis | 6 |
|  | Antibiotic therapy | 6 |
|  | Cardiac failure chronic | 5 |
|  | Depression | 4 |
|  | Pain | 4 |
|  | Hypertension | 4 |
|  | Pruritus | 1 |
|  | Restlessness | 1 |
|  | Nausea | 1 |
|  | Benign prostatic hyperplasia | 1 |
|  | Radiation pneumonitis | 1 |
|  | Antifungal prophylaxis | 1 |
| **Nivolumab Monotherapy** |  |  |
|  | Hypertension | 58 |
|  | Atrial fibrillation | 22 |
|  | Gastroesophageal reflux disease | 21 |
|  | HIV infection | 12 |
|  | Hyperuricaemia | 7 |
|  | Hypothyroidism | 7 |
|  | Prophylaxis | 6 |
|  | Vitamin supplementation | 6 |
|  | Cerebrovascular accident prophylaxis | 6 |
|  | Cardiovascular event prophylaxis | 6 |
|  | Nausea | 6 |
|  | Antifungal prophylaxis | 6 |

**Note:** Non-cancer indication proxies were extracted from FAERS drug and indication records after excluding oncologic indications and target renal or pancreatic adverse event terms. These variables provide descriptive clinical context and should not be interpreted as confirmed baseline comorbidities.

**Supplementary Table 4. Report-level serious outcome codes among reports with renal and/or pancreatic adverse events by treatment cohort**

| **Outcome** | **Combination**  **(n = 60)** | **Nivolumab Monotherapy**  **(n = 156)** | **P value** |
| --- | --- | --- | --- |
| Death, n (%) | 7 (11.7%) | 21 (13.5%) | 0.824 |
| Life-threatening, n (%) | 7 (11.7%) | 29 (18.6%) | 0.308 |
| Hospitalization, n (%) | 32 (53.3%) | 106 (67.9%) | 0.058 |
| Disability, n (%) | 1 (1.7%) | 11 (7.1%) | 0.186 |

**Note:** Values are presented as n (%). P values were calculated using Fisher’s exact test. Outcomes are not mutually exclusive because one report may contain multiple serious outcome codes. The denominator includes reports with at least one renal or pancreatic adverse event in each treatment cohort.
